# Aberrant Gene Promoter Methylation Associated with Sporadic Multiple Colorectal Cancer

**DOI:** 10.1371/journal.pone.0008777

**Published:** 2010-01-19

**Authors:** Victoria Gonzalo, Juan José Lozano, Jenifer Muñoz, Francesc Balaguer, Maria Pellisé, Cristina Rodríguez de Miguel, Montserrat Andreu, Rodrigo Jover, Xavier Llor, M. Dolores Giráldez, Teresa Ocaña, Anna Serradesanferm, Virginia Alonso-Espinaco, Mireya Jimeno, Miriam Cuatrecasas, Oriol Sendino, Sergi Castellví-Bel, Antoni Castells, for the Gastrointestinal Oncology Group of the Spanish Gastroenterological Association

**Affiliations:** 1 Department of Gastroenterology, Institut de Malalties Digestives i Metabòliques, Hospital Clínic, Centro de Investigación Biomédica en Red en el Área temática de Enfermedades Hepáticas y Digestivas (CIBERehd), Institut d'investigacions Biomèdiques August Pi i Sunyer (IDIBAPS), University of Barcelona, Barcelona, Catalonia, Spain; 2 Plataforma de Bioinformática, Centro de Investigación Biomédica en Red en el Área temática de Enfermedades Hepáticas y Digestivas (CIBERehd), Barcelona, Catalonia, Spain; 3 Gastroenterology Department, Hospital del Mar, Barcelona, Catalonia, Spain; 4 Gastroenterology Department, Hospital General Universitario de Alicante, Alicante, Spain; 5 Section of Digestive Diseases and Nutrition, University of Illinois at Chicago, Chicago, Illinois, United States of America; 6 Preventive Medicine and Epidemiology Unit, Hospital Clínic, Centro de Investigación Biomédica en Red en el Área temática de Enfermedades Hepáticas y Digestivas (CIBERehd), Institut d'investigacions Biomèdiques August Pi i Sunyer (IDIBAPS), University of Barcelona, Barcelona, Catalonia, Spain; 7 Department of Pathology, Centre de Diagnòstic Biomèdic; Hospital Clínic, Centro de Investigación Biomédica en Red en el Área temática de Enfermedades Hepáticas y Digestivas (CIBERehd), Institut d'investigacions Biomèdiques August Pi i Sunyer (IDIBAPS), University of Barcelona, Barcelona, Catalonia, Spain; Ohio State University Medical Center, United States of America

## Abstract

**Background:**

Colorectal cancer (CRC) multiplicity has been mainly related to polyposis and non-polyposis hereditary syndromes. In sporadic CRC, aberrant gene promoter methylation has been shown to play a key role in carcinogenesis, although little is known about its involvement in multiplicity. To assess the effect of methylation in tumor multiplicity in sporadic CRC, hypermethylation of key tumor suppressor genes was evaluated in patients with both multiple and solitary tumors, as a proof-of-concept of an underlying epigenetic defect.

**Methodology/Principal Findings:**

We examined a total of 47 synchronous/metachronous primary CRC from 41 patients, and 41 gender, age (5-year intervals) and tumor location-paired patients with solitary tumors. Exclusion criteria were polyposis syndromes, Lynch syndrome and inflammatory bowel disease. DNA methylation at the promoter region of the *MGMT*, *CDKN2A*, S*FRP1*, *TMEFF2*, *HS3ST2 (3OST2)*, *RASSF1A* and *GATA4* genes was evaluated by quantitative methylation specific PCR in both tumor and corresponding normal appearing colorectal mucosa samples. Overall, patients with multiple lesions exhibited a higher degree of methylation in tumor samples than those with solitary tumors regarding all evaluated genes. After adjusting for age and gender, binomial logistic regression analysis identified methylation of *MGMT2* (OR, 1.48; 95% CI, 1.10 to 1.97; p = 0.008) and *RASSF1A* (OR, 2.04; 95% CI, 1.01 to 4.13; p = 0.047) as variables independently associated with tumor multiplicity, being the risk related to methylation of any of these two genes 4.57 (95% CI, 1.53 to 13.61; p = 0.006). Moreover, in six patients in whom both tumors were available, we found a correlation in the methylation levels of *MGMT2* (r = 0.64, p = 0.17), *SFRP1* (r = 0.83, 0.06), *HPP1* (r = 0.64, p = 0.17), *3OST2* (r = 0.83, p = 0.06) and *GATA4* (r = 0.6, p = 0.24). Methylation in normal appearing colorectal mucosa from patients with multiple and solitary CRC showed no relevant difference in any evaluated gene.

**Conclusions:**

These results provide a proof-of-concept that gene promoter methylation is associated with tumor multiplicity. This underlying epigenetic defect may have noteworthy implications in the prevention of patients with sporadic CRC.

## Introduction

Colorectal cancer (CRC) is a relevant public health problem since it represents the second most common malignant tumor and also the second leading cause of cancer death in Western countries. Inheritance constitutes the underlying cause in up to one third of all CRC cases, with highly penetrant and well-defined hereditary disorders, i.e. adenomatous and hamartomatous polyposis and Lynch syndrome, representing 3–5% of the total CRC burden [1]. In such conditions, presence of a germline mutation in the causative gene (i.e. *APC*, *MYH*, *LKB1*, *SMAD4*, *BMPR1A*, *PTEN*, *MLH1*, *MSH2*, *MSH6* and *PMS2*) [1], [2], predisposes these individuals to the development of multiple colorectal neoplasms. Indeed, whereas familial adenomatous polyposis represents the paradigm of tumor multiplicity, presence of either synchronous or metachronous CRC is also one of the most common clinical criteria for suspecting Lynch syndrome [3].

Besides the above-mentioned inherited conditions, tumor multiplicity is also a frequent observation in patients with CRC who are not apparently predisposed to these neoplasms on the basis of their genetic background. In fact, synchronous and metachronous colorectal adenomas are reported in up to 30% and 48% of patients with sporadic CRC, respectively, while the corresponding figures for carcinoma are 4% and 9%, respectively [4], [5]. In this setting, evident familial cancer aggregation or distinctive personal characteristics are not openly distinguished, and although a generalized cellular or molecular disorder in the entire colorectal mucosa may be suspected, the underlying pathogenic mechanism remains elusive.

A field effect underlying colorectal carcinogenesis is a well recognized situation in patients with inflammatory bowel disease, a premalignant condition with an increased cumulative risk for developing CRC associated with early age of onset, disease duration, and extent and severity of inflammation [6], [7]. The precise mechanism by which chronic colonic mucosal inflammation causes malignancy in this context is poorly understood, although it is supposed to be related to a failure in regulatory mechanisms during cell division. Chronic inflammation leads to the release of free radicals from leucocytes and macrophages, and these reactive oxygen species can drive carcinogenesis by causing DNA damage [8]. Since in most cases DNA damage leads to inactivation of tumor suppressor genes, the concept of “field effect” could be better designated as “field defect”. Putative involvement of such a field defect in sporadic CRC, however, has not been satisfactorily established so far.

Sporadic CRC arises as a consequence of the accumulation of genetic and epigenetic alterations that transform colonic epithelial cells into colon adenocarcinoma cells [9]. The loss of genomic stability and resulting gene alterations are key molecular pathogenic steps that occur early in tumorigenesis: they permit the acquisition of a sufficient number of alterations in tumor suppressor genes and oncogenes that transform cells and promote tumor progression. Analogous to genomic instability, epigenetic instability results in the aberrant methylation of tumor suppressor genes [9]. In fact, epigenetic tumor suppressor gene silencing has commonly been involved in all types of human tumors, including CRC [10]. Aberrant cytosine methylation plays a preeminent role in cell transformation when it affects genes that safeguard genome instability. This epigenetic change can also be detected in precancerous lesions and seemingly normal peritumor tissues [11], [12], [13], [14], [15], thus suggesting its potential involvement in the initial carcinogenetic process. This putative field defect associated with gene promoter hypermethylation in normal appearing colorectal mucosa has been suggested with respect to the *MGMT* gene [12], as well as *ERα*, *MYOD*, *P16(INK4A)*, *MLH1*, *TIMP3* and *DAPK* [13].

Considering that hypermethylation of promoter regions in tumor suppressor genes could be observed in normal appearing colorectal mucosa, we hypothesized that this phenomenon would be especially relevant in patients who developed multiple CRC. Therefore, the aim of this study was to evaluate methylation patterns of genes involved in colorectal carcinogenesis through this mechanism in both tumor tissue and normal appearing colorectal mucosa samples of patients with multiple and solitary CRC, as a proof-of-concept of a putative underlying epigenetic defect associated with tumor multiplicity.

## Materials and Methods

### Patients

We examined a total of 47 synchronous/metachronous primary CRC from 41 patients (36 synchronous, 4 metachronous and one both) and 41 gender, age (5-year intervals) and tumor location-paired patients with solitary tumors. Control patients with solitary tumors were recruited in the EPICOLON project, a prospective, multicenter, nation-wide, population-based cohort (n = 1,222) [16] and randomly selected among those with no previous CRC and with a minimum follow-up of 5 year after the diagnosis of cancer in which regular colonoscopy surveillance did not identify any additional lesion. Regarding patients with multiple CRC, 31 were also recruited in the EPICOLON project and 10 additional patients at the Endoscopy Unit of the Hospital Clínic of Barcelona between June 2007 and May 2008. There were no differences with respect to the clinicopathological characteristics of both sets of patients with multiple lesions (data not shown). Exclusion criteria for the present study were colorectal polyposis syndromes, Lynch syndrome and personal history of inflammatory bowel disease. Demographic, clinical and tumor-related characteristics of patients included in the study are summarized in Table 1. The study was approved by the institutional Ethics Committee of each participating hospital, and written informed consent was obtained from all patients. Members of the EPICOLON project are listed in Note S1.

**Table 1:** Characteristics of patients included in the study.

	Multiple CRC (n = 41)	Solitary CRC (n = 41)	P value^1^
Age (yrs.)^2^	74.3±8.2	74.9±9.1	0.97
Gender –no. (%)			0.82
male	28 (68.3%)	26 (63.4%)	
female	13 (31.7%)	15 (36.6%)	
Tumor location –no. (%)^3^			1.0
right	25 (61.0%)	25 (61.0%)	
left	16 (39.0%)	16 (39.0%)	
Tumor stage –no. (%)^3^			0.82
I	4 (9.8%)	5 (12.1%)	
II	15 (36.6%)	19 (46.3%)	
III	13 (31.7%)	12 (29.3%)	
IV	7 (17.1%)	5 (12.2%)	
Tumor multiplicity –no. (%)			-
synchronous	36 (87.8%)	NA	
metachronous	4 (9.8%)	NA	
both	1 (2.4%)	NA	
Synchronous adenoma –no. (%)	25 (61.0%)	1 (2.4%)	0.001
Personal history of other neoplasms –no. (%)^4^	1 (2.4%)	1 (2.4%)	1.0
Family history of colorectal cancer –no. (%)^5^	5 (12.2%)	7 (17.1%)	0.76
Family history of other neoplasms –no. (%)^5^	2 (4.9%)^6^	5 (12.2%)	0.43
endometrial	2	1	
gastric	1	3	
ovary	-	1	
Tumor DNA mismatch repair deficiency –no. (%)	3 (7.3%)	2 (4.9%)	0.64

CRC, colorectal cancer; NA, not applied.

1 Qualitative variables were compared by the Fisher's exact test; continuous variables were compared by the Mann-Whitney U's test.

2 Results expressed as mean ± standard deviation.

3 In patients with multiple tumors, characteristics were referred to the most advanced lesion.

4 Other neoplasms included small bowel and ovary, respectively.

5 Family history was referred to first degree relatives.

6 One patient had both endometrial and gastric cancer.

Frozen tumor and corresponding normal-appearing, peritumor colorectal mucosa tissues were obtained either at surgery or endoscopy from all patients, and immediately stored at −80°C until use. In patients with multiple lesions, tissue sample was obtained from at least one tumor (the most advanced one or the largest when multiple tumors had the same tumor stage).

### DNA Isolation and Bisulfite Treatment

Frozen samples were thawed and genomic DNA was isolated using the QIAamp DNA Mini Kit® (Qiagen, Valencia, CA) according to the manufacturer's instructions. Bisulfite treatment was carried out on genomic DNA using the EZ DNA Methylation-Gold Kit® (Zymo Research, Orange, CA) according to the manufacturer's protocol with minor modifications detailed below [17]. This procedure integrated the DNA denaturation and bisulfite conversion into one-step, using temperature denaturation to replace chemical denaturation with sodium hydroxide, and it was based on a three-step reaction process between cytosine and sodium bisulfite that converts unmethylated cytosines into uracils. An amount of 250 ng of genomic DNA isolated from each tumor or normal tissue sample was used per reaction, and a volume of 15 µl was employed for each bisulfited DNA to be eluted. The resulted DNA was used for PCR amplification or stored at −80°C.

### Quantitative Methylation Specific PCR

After bisulfite conversion, duplicates of 0.5 µl of each bisulfited DNA were amplified by the MethyLight technique, a previously described fluorescence-based quantitative real-time PCR, highly specific, sensitive and reproducible assay [18]. Locus specific PCR primers and probes for seven tumor suppressor genes –*MGMT1* (minimal promoter), *MGMT2* (enhancer region), *CDKN2A*, *SFRP1*, *TMEFF2*, *HS3ST2 (3OST2)*, *RASSF1A* and *GATA4*– were specifically designed for bisulfited-converted DNA sequences and located at each gene promoter region. These genes were chosen for their involvement in colorectal carcinogenesis through methylation-driven silencing and evidence of some degree of hypermethylation in normal-appearing, peritumor colorectal mucosa counterpart (Table 2). In that sense, it is important to emphasize that genes proposed as markers of the CpG island methylator phenotype which, by definition, are almost exclusively methylated in cancer tissue were avoided. Primer and probes used for bisulfited DNA sequences are listed on Table S1. Fully unmethylated and fully *Sss*l-methylated DNA were employed initially as 0 and 100% methylated references to test amplification results, and methylated DNA was further used as calibrator for all tested samples. *ALUC4* gene was used as endogenous reference to normalize for the amount of input DNA [19]. The MethyLight reactions were performed on a 7300 Real Time PCR System (Applied Biosystems, Foster City, CA) with a final volume of 12.5 µl containing 900 nM of each primer and 250 nM of the corresponding probe. The PCR conditions were: 95°C for 10 minutes, followed by 40 cycles at 92°C for 15 seconds and 58°C for 1 minute, as it was previously described [18].

**Table 2:** Genes evaluated in the study.

Gene abbreviation	Gene name	GenBank accession no.	Reference
*MGMT*	O6-methylguanine-DNA-methyltransferase	NM_002412	[12], [14], [41], [42], [43]
*CDKN2A (p16)*	Cyclin-dependent kinase inhibitor 2 A	NM_000077	[13], [44], [45]
*SFRP1*	Secreted frizzled-related protein 1	NM_003012	[46], [47]
*TMEFF2*	Transmembrane protein with EGF-like and two follistatin-like domains 2	NM_016192	[48], [49]
*HS3ST2 (3OST2)*	Heparan sulfate (glucosamine) 3-O-sulfotransferase 2	NM_006043	[36], [50]
*RASSF1A*	Ras association (RalGDS/AF-6) domain family member 1	AF132675	[33], [34], [35], [36], [37]
*GATA4*	GATA binding protein 4	NM_002052	[51]

Each measurement in a given sample was performed in duplicate for both tested and endogenous genes, and the threshold cycle (C_t_) –the fractional number at which the amount of amplified target reached a fixed threshold– was determined. The standard deviation in sample duplicates was always below 0.2. Relative amounts of both genes were also normalized to commercial 100% methylated DNA (Zymo Research, Orange, CA) acting as calibrator to allow comparison across all tested samples. The comparative C_t_ method [20], also known as the 2^−ΔΔCt^ method, was calculated from
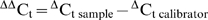
where ^Δ^C_t, sample_ was the tested genes C_t_ value for any sample normalized to *ALUC4*, and ^Δ^C_t, calibrator_ was the tested genes C_t_ value for the calibrator also normalized to *ALUC4*. The result derived from the ^ΔΔ^C_t_ ×100 corresponds to percentage of methylated reference (PMR), which indicates the percentage of fully methylated molecules at a specific locus [21].

Investigators performing the methylation specific PCR real-time experiments were blinded to the clinical characteristics of patients (i.e. tumor multiplicity).

### Evaluation of Tumor Mismatch Repair Deficiency

Tumor mismatch repair deficiency was evaluated by both immunostaining and microsatellite instability testing. Immunohistochemical analysis included evaluation of MSH2 (anti-MSH2, Oncogene Research Products, Boston, MA), MLH1 (anti-MLH1, PharMingen, San Diego, CA) and MSH6 (anti-MSH6, BD Transduction Laboratories, San José, CA), as described elsewhere [16]. Tumor cells were judged to be negative for protein expression only if they lacked staining in a sample in which normal colonocytes and stroma cells were stained. If no immunostaining of normal tissue could be demonstrated, the results were considered unreliable. Microsatellite instability was assessed using the 5-marker panel proposed by the National Cancer Institute and/or the pentaplex of mononucleotide repeats, as described elsewhere [22].

### Statistical Analysis

Comparison of methylation degree between multiple and solitary CRC patients was performed qualitatively where methylation positivity was set as PMR ≥4, as previously validated [23]. Since information regarding methylation in normal appearing colorectal mucosa was limited, the analysis in this setting was performed according to both a ≥4 PMR cut-off and an additional, arbitrarily chosen ≥1 PMR cut-off in order to ascertain any potential minor effect. The analysis was performed using the Fisher's exact test. Correlation between methylation levels of tumor pairs was analyzed by Spearman correlation analysis.

Comparison between patients with multiple and solitary tumors regarding the methylation degree in both tumor and normal appearing colorectal mucosa samples was also performed using binomial logistic regression, both unadjusted and adjusted for age and gender. Furthermore, we tested the independent effect of gene methylation on tumor multiplicity by including all evaluated genes in the binomial logistic regression model, along with age and gender. These variables were “pruned” using an automated stepwise procedure for optimizing the Akaike information criterion [24]. Multiplicative interactions were tested for each pair of genes independently associated with tumor multiplicity by including both main effects and an interaction term (a product of two main effects) in the logistic regression model. Finally, we tested the cumulative effects of methylated genes on tumor multiplicity by counting the number of selected genes independently associated with this phenomenon in each subject. The odds ratio for tumor multiplicity for patients carrying any combination of the selected methylated genes was estimated by comparing them with patients carrying none of these genes with the use of logistic regression analysis. Statistical analyses were carried out using “R” (R Core Development team, http://www.R-project.org).

Continuous variables were expressed as mean ± standard deviation. All p values were two sided. A p value of less than 0.05 was considered to indicate a statistically significant difference.

## Results

Forty-one patients with either synchronous or metachronous CRC, and 41 gender, age and tumor location-paired patients with solitary tumors constituted the basis of this study. As it is shown in Table 1, both groups of patients were similar with respect to any demographic, clinical and tumor-related characteristics, except for the presence of synchronous colorectal adenomas.

### Gene Promoter Methylation in Tumor Samples

Comparison of gene promoter methylation in tumor samples from patients with multiple and solitary CRC is depicted in Table 3. Overall, patients with multiple lesions exhibited a higher degree of methylation in tumor samples than those with solitary tumors regarding all evaluated genes. The proportion of tumors exhibiting gene promoter hypermethylation was significantly higher in patients with multiple lesions than in those with solitary CRC with respect to *MGMT2* (40.4% *vs.* 14.6%, respectively; p = 0.009) and *RASSF1A* (17.0% *vs.* 0%, respectively; p = 0.006) (Table 3).

**Table 3:** Gene promoter methylation in tumor samples.

	Multiple CRC (n = 47)	Solitary CRC (n = 41)	P value^1^
***MGMT1*** **-Mp**			0.21
methylated -no. (%)	8 (17.0)	3 (7.3)	
unmethylated-no. (%)	39 (83.0)	38 (92.7)	
***MGMT2*** **-Enh**			0.009
methylated -no. (%)	19 (40.4)	6 (14.6)	
unmethylated-no. (%)	28 (49.6)	35 (85.4)	
***CDKN2A***			0.08
methylated -no. (%)	10 (21.3)	3 (7.3)	
unmethylated-no. (%)	37 (78.7)	38 (92.7)	
***SFRP1***			0.53
methylated -no. (%)	42 (89.4)	34 (82.9)	
unmethylated-no. (%)	5 (10.6)	7 (17.1)	
***TMEFF2***			0.66
methylated -no. (%)	20 (42.6)	15 (36.6)	
unmethylated-no. (%)	27 (57.4)	36 (63.4)	
***HS3ST2 (3OST2)***			0.46
methylated -no. (%)	37 (78.7)	29 (70.7)	
unmethylated-no. (%)	10 (21.3)	12 (29.3)	
***RASSF1A***			0.006
methylated -no. (%)	8 (17.0)	- (-)	
unmethylated-no. (%)	39 (83.0)	- (-)	
***GATA4***			0.057
methylated -no. (%)	38 (80.9)	25 (60.9)	
unmethylated-no. (%)	9 (19.1)	16 (39.1)	

CRC, colorectal cancer.

1 Variables were compared by the Fisher's exact test.

Estimation of the risk of tumor multiplicity associated with gene promoter methylation in tumor samples is shown in Table 4. After adjusting for age and gender, binomial logistic regression analysis indicated that methylation of promoter regions of the *MGMT1* locus (odds ratio (OR), 1.57; 95% confidence interval (CI), 1.01 to 2.43; p = 0.04), *MGMT2* locus (OR, 1.50; 95% CI, 1.14 to 1.96; p = 0.003), and *RASSF1A* gene (OR, 2.02; 95% CI, 1.03 to 3.93; p = 0.03) were associated with an increased risk of developing multiple CRC (Table 4).

**Table 4:** Risk of tumor multiplicity based on gene promoter methylation in tumor^1^.

	Unadjusted			Adjusted^2^		
	OR	95% CI	P value	OR	95% CI	P value
***MGMT1*** **-Mp**						
unmethylated	1 (ref)	-		1 (ref)	-	
methylated	1.58	1.02–2.44	0.03	1.57	1.01–2.43	0.04
***MGMT2*** **-Enh**						
unmethylated	1 (ref)	-		1 (ref)	-	
methylated	1.48	1.14–1.93	0.003	1.50	1.14–1.96	0.003
***CDKN2A***						
unmethylated	1 (ref)	-		1 (ref)	-	
methylated	1.23	0.92–1.65	0.15	1.23	0.92–1.65	0.16
***SFRP1***						
unmethylated	1 (ref)	-		1 (ref)	-	
methylated	1.06	0.87–1.29	0.51	1.06	0.87–1.29	0.55
***TMEFF2***						
unmethylated	1 (ref)	-		1 (ref)	-	
methylated	1.13	0.89–1.43	0.29	1.13	0.89–1.44	0.29
***HS3ST2 (3OST2)***						
unmethylated	1 (ref)	-		1 (ref)	-	
methylated	1.12	0.90–1.38	0.29	1.12	0.90–1.40	0.29
***RASSF1A***						
unmethylated	1 (ref)	-		1 (ref)	-	
methylated	1.95	1.01–3.74	0.04	2.02	1.03–3.93	0.03
***GATA4***						
unmethylated	1 (ref)	-		1 (ref)	-	
methylated	1.10	0.89–1.35	0.34	1.10	0.89–1.36	0.34

OR, odds ratio; 95% CI, 95% confidence interval.

1 Binomial logistic regression analysis.

2 Adjusted by age and gender.

The adjusted multivariate logistic regression analysis identified methylation of the *MGMT2* locus (OR, 1.48; 95% CI, 1.10 to 1.97; p = 0.008) and *RASSF1A* gene (OR, 2.04; 95% CI, 1.01 to 4.13; p = 0.047) as variables independently associated with tumor multiplicity. In addition, when the product of these two variables was added to the regression model, this interaction term was not selected (OR, 0.88; 95% CI, 0.67 to 1.16; p = 0.37). Lastly, when the cumulative effects of methylated genes was evaluated, the risk of tumor multiplicity associated with methylation of any of these two selected genes was 4.57 (95% CI, 1.53 to 13.61; p = 0.006), with no significant increase when both genes were simultaneously methylated (OR, 2.31; 95% CI, 0.00 to undetermined; p = 0.99).

**Figure 1:**
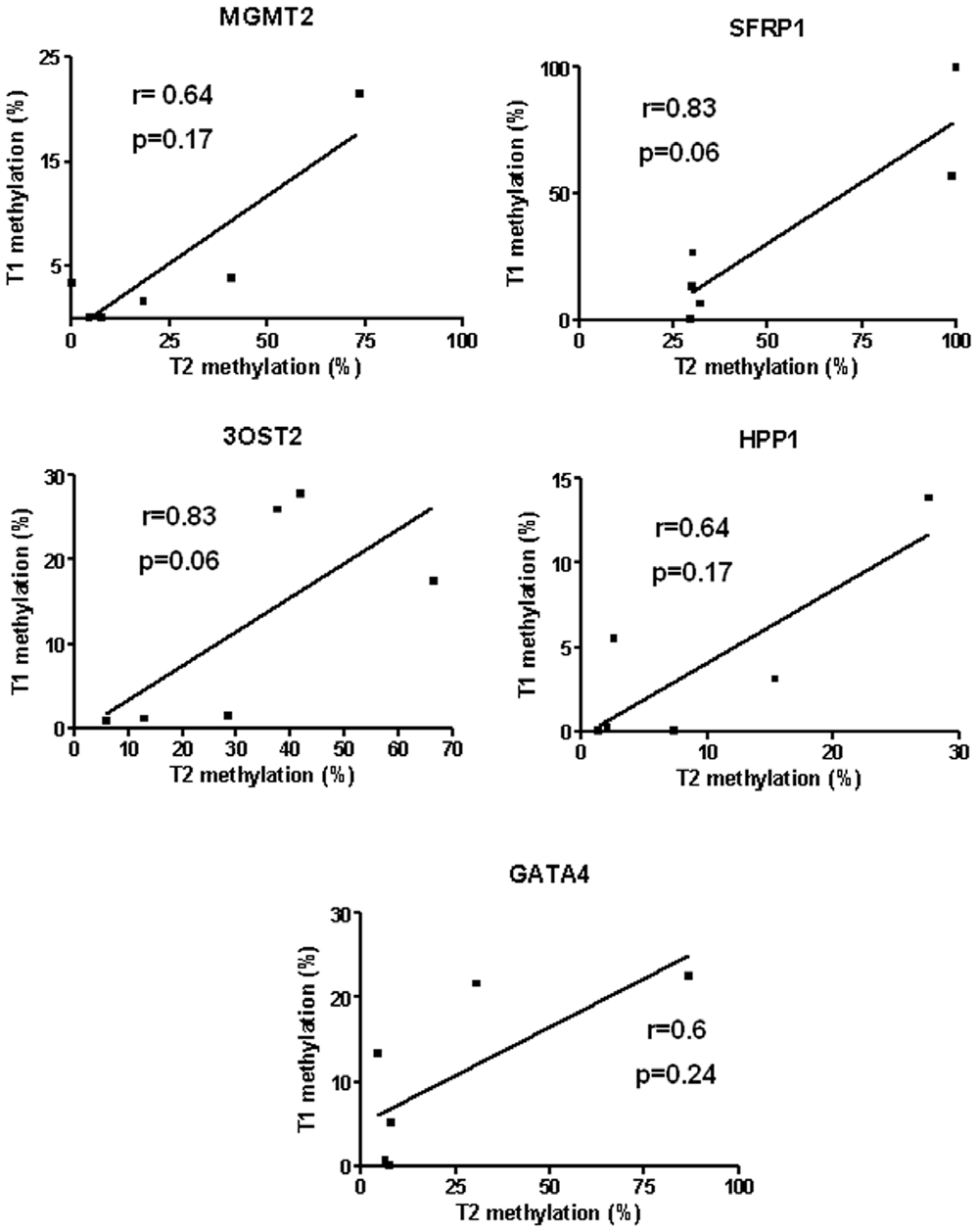
Correlation in methylation levels of MGMT2, SFRP1, 3OST2, HPP1 and GATA4 in 6 tumor pairs from patients with multiple tumors in whom both lesions were available. Results are expressed as percentage of methylation based on PMR.

Finally, we analyzed the correlation in methylation levels in the subset of six patients with multiple tumors in whom both tumors were available for analysis (Figure 1). This analysis showed a non-significant correlation in the methylation levels of *MGMT2* (r = 64, p = 0.17), *SFRP1* (r = 0.83, 0.06), *HPP1* (r = 0.64, p = 0.17), *3OST2* (r = 0.83, p = 0.06), and *GATA4* (r = 0.6, p = 0.24). *MGMT1* and *CDKN2A* did not show evidence of concordance between tumors in the same patient (r = −0.05, p = 0.91; r = −0.09, p = 0.91, respectively), and *RASSF1A* was rarely methylated in these tumors, which precluded a proper correlation analysis.

### Gene Promoter Methylation in Normal Appearing Colorectal Mucosa Samples

Methylation in normal appearing colorectal mucosa from patients with multiple and solitary CRC showed no relevant difference in any evaluated gene (Table 5). In order to ascertain any potential minor effect, the analysis was repeated using a ≥1 PMR cut-off (Table 5). In this second analysis, no consistent methylation pattern was observed, with some genes showing hypermethylation (i.e. *MGMT1*, *MGMT2* and *RASSF1A*) and others hypomethylation (i.e. *SFRP1*, *TMEFF2* and *GATA4*) in patients with multiple lesions. None of these differences were statistically significant (Table 5).

**Table 5:** Gene promoter methylation in normal appearing colorectal mucosa samples.

	Multiple CRC (n = 47)	Solitary CRC (n = 41)	P value^1^
***MGMT1*** **-Mp**			
PMR≥4 –no. (%)	- (-)	- (-)	-
PMR≥1 –no. (%)	1 (2.1)	- (-)	-
***MGMT2***-Enh			
PMR≥4 –no. (%)	- (-)	1 (2.4)	0.46
PMR≥1 –no. (%)	3 (6.4)	1 (2.4)	0.62
***CDKN2A***			
PMR≥4 –no. (%)	- (-)	- (-)	-
PMR≥1 –no. (%)	- (-)	- (-)	-
***SFRP1***			
PMR≥4 –no. (%)	26 (55.3)	27 (65.9)	0.38
PMR≥1 –no. (%)	36 (76.6)	35 (85.4)	0.41
***TMEFF2***			
PMR≥4 –no. (%)	- (-)	1 (2.4)	0.47
PMR≥1 –no. (%)	3 (6.4)	8 (19.5)	0.10
***HS3ST2 (3OST2)***			
PMR≥4 –no. (%)	9 (19.1)	14 (34.1)	0.14
PMR≥1 –no. (%)	36 (76.6)	32 (78.0)	1.0
***RASSF1A***			
PMR≥4 –no. (%)	- (-)	- (-)	-
PMR≥1 –no. (%)	1 (2.1)	- (-)	-
***GATA4***			
PMR≥4 –no. (%)	9 (19.1)	12 (29.3)	0.32
PMR≥1 –no. (%)	29 (61.7)	30 (73.2)	0.26

CRC, colorectal cancer; PMR, percentage of methylated reference.

1 Variables were compared by the Fisher's exact test.

## Discussion

Results of this study demonstrate that tumors from patients with synchronous and metachronous CRC exhibit a higher degree of methylation than those from patients with solitary lesions. Tumor hypermethylation of the *MGMT* gene enhancer region and the *RASSF1A* gene promoter region were identified as variables independently associated with a five-fold increased risk of developing multiple lesions. Moreover, we found similar methylation patterns in tumor pairs from the same patient. Overall, these observations provide a proof-of-concept of an epigenetic defect mediated by gene promoter hypermethylation which favor tumor multiplicity in sporadic CRC.

Strengths of this study rely on the fact that it was carried out on a general population through a prospective, multicenter, nation-wide study in which unselected and consecutive patients with CRC were included regardless of their personal and familial characteristics; previous genetic characterization performed in the context of the EPICOLON project allowed an adequate identification and subsequent exclusion of patients with inherited disorders (i.e. colorectal polyposis, Lynch syndrome and MYH-associated CRC) [16], [22], [25], [26], [27], [28], in whom a specific and well-defined molecular mechanism justifies tumor multiplicity; it represents the largest series of patients with multiple lesions evaluated so far for tumor methylation, as well as the first study in which a control group of patients with a solitary lesion was included with adequate stratification for gender, age and tumor location; and finally, quantitative methylation specific PCR was performed in both tumor sample and paired normal appearing colorectal mucosa, and data analyzed in a blinded fashion.

We are aware, however, of some limitations of this study. First, RNA samples were not available to perform parallel expression analyses and verify the biological significance of gene promoter methylation. Nevertheless, there is a large body of evidence that MethyLight assays provide an excellent correlation between promoter methylation and gene silencing in similar tumor settings [11], [29]. More uncertainty exists, however, with respect to the value of these results in non-neoplastic tissues. Although it has been suggested that the epigenetic signature of cancers may have early-stage, normal-tissue counterparts potentially involved in the initiation of carcinogenetic process [14], it is still unclear if the same cut-off of methylation used for tumor samples (i.e. PMR ≥4) can be employed in non-neoplastic tissues. In order to overcome this limitation, results obtained in normal appearing colorectal mucosa were analyzed using two different cut-off levels. Second, this study represents a candidate-gene, hypothesis-driven investigation in which a reduced number of genes were chosen on the basis of previous information demonstrating their involvement in colorectal carcinogenesis through methylation-mediated gene silencing, and evidence of a decreasing degree of hypermethylation among tumor, peritumor normal appearing colorectal mucosa, and normal colorectal mucosa from non-tumor individuals. The main purpose of this approach was to provide a proof-of-concept of the potential involvement of gene promoter hypermethylation in tumor multiplicity rather than identifying the epigenetic signature underlying this process. To reach this latter goal, high-throughput techniques with genome wide capability are required, an approach currently ongoing in our laboratory. Third, evaluation of normal-appearing colorectal mucosa was limited to the peritumor area in the vast majority of cases, since most samples were obtained from surgical specimens. This aspect precludes generalizing the results obtained in seemingly normal mucosa to the entire colon. Indeed, striking colon segment-specific differences in the prevalence of methylation of some genes (i.e. *MLH1* and *MGMT*) have been observed [14]. How this scatter pattern would affect the potential use of methylation analysis in CRC risk assessment and, consequently, putative methylation-driven screening and surveillance strategies, is currently being evaluated.

A field defect mediated by *MGMT* gene promoter methylation has been previously suggested [12]. In that seminal study, hypermethylation of the *MGMT* gene was observed in 46% of tumors as well as in 50% of normal appearing colorectal mucosa samples of patients in whom *MGMT* promoter methylation was found in the corresponding tumor. In another study, participation of DNA methylation in five CIMP-specific gene promoters, including *MGMT*, was also evaluated in six synchronous carcinoma pairs [30]. In this study, it was observed that while some tumor pairs showed discordant methylation patterns, others showed similar, but not exactly identical, profiles of promoter methylation, suggesting that epigenetic alterations in synchronous CRC likely have both random and nonrandom components [30]. Recently, Konishi *et al.* found significant differences in methylation between multiple tumors compared to solitary lesions for *MGMT* (26.5% *vs.* 17.3%; p<0.05) and *p14* (16.1% *vs.* 9.3%; p<0.05) [31]. Interestingly, these authors found a significant correlation for methylation of different genes, including *MGMT*, between tumor pairs of the same site (proximal *vs.* distal). Unfortunately, this interesting issue could only be partially addressed in our investigation since, because of the design of the EPICOLON project, only one tumor sample was collected from most patients with synchronous CRC, thus limiting this pair-wise comparison to 6 patients. Although the positive correlations for *MGMT2* did not reach statistical significance (probably due the low number of paired tumors available), our results are consistent with the data obtained by Konishi *et al.* [31], supporting the hypothesis that patients with multiple tumors show concordant methylation in their tumor tissues. Very recently, in a seminal publication, LINE-1 methylation levels were significantly correlated in 10 synchronous CRC pairs, thus reinforcing the hypothesis of a field effect [32].

Methylation-associated inactivation of *RASSF1A* has been frequently observed in several human malignancies including sporadic CRC [33], [34], [35], [36], [37]. Indeed, tumor promoter hypermethylation of *RASSF1A* occurs in approximately 20% of CRC, and it seems to exist a mutually exclusive relationship with the presence of *KRAS* mutations [34], [35]. Interestingly, in tumors with mismatch repair deficiency, no significant differences were observed in the frequency of *RASSF1A* methylation between unstable sporadic CRC and tumors associated with Lynch syndrome [37]. The above mentioned results [32], along with the demonstration of *RASSF1A* methylation in tumor samples from patients with multiple lesions, and the lack of differences in other factors predisposing to tumor multiplicity (i.e. family history) favor the hypothesis of an underlying epigenetic defect. However, whether this methylation-driven gene silencing mechanism represents a potential field effect due to an unidentified molecular alteration in normal mucosa or the expression of pre-existing multiple hyperplastic polyps from which CRC arises through the serrated pathway [38], as it has been recently suggested [32], remains unknown.

As it was mentioned, aberrant methylation of some CpG islands has been seen in normal appearing colorectal mucosa. In one study [13], this phenomenon was demonstrated for the *ERα* and *MYOD* genes, as well as for the *P16(INK4A)*, *MLH1*, *TIMP3* and *DAPK* genes at a lower level. Interestingly, some gene polymorphisms were associated with a lower methylation of the CpG islands examined, thus suggesting that genetic factors can influence this epigenetic alteration in normal colorectal mucosa [13]. The physiological conditions associated with aberrant promoter methylation in seemingly normal colorectal mucosa have also been recently evaluated with respect to two DNA-repair genes, *MLH1* and *MGMT* [14]. In that study, samples from males showed no consistent patterns for either promoter, but the prevalence of *MLH1* and *MGMT* methylation increased significantly with age, particularly in the right colon, and were consistent with current epigenetic profiles of CRC subsets. Similar results were obtained in a third study, in which methylation frequencies of colorectal adenomas were intermediate between CRC and seemingly normal mucosa [36]. Regarding the role of methylation in normal appearing colonic mucosa, Konishi *et al.* recently evaluated the methylation status of several genes (*MINT1*, *MINT2*, *MINT31*, *MLH1*, *p14*, *p16*, *MGMT*, and *ESR1*) in the tumor-adjacent normal mucosa from patients with multiple and solitary tumors, and found no significant differences between both groups [31]. The methylation levels for all genes, except for *MGMT* and *ESR1*, were at a vey low level. It is important to note that the low level of methylation in normal appearing colorectal mucosa observed in our study, in a similar manner as in others [13], [31], may be due to the circumscription of this phenomenon to limited areas (aberrant crypt foci, for instance) rather than a spread, diffuse alteration throughout the colon [39]. Another possibility would be that this molecular event may affect some specific cellular subtypes, the recent identified colon cancer tumor-initiating cells being the most attractive candidate [40].

In conclusion, results of this study demonstrate that sporadic CRC multiplicity is associated with gene promoter methylation. If further investigations were able to identify the epigenetic signature associated with tumor multiplicity and/or provide further evidence of a potential field defect, a new approach to CRC risk assessment and prevention would be available.

## Supporting Information

Note S1 Investigators from the Gastrointestinal Oncology Group of the Spanish Gastroenterological Association who participated in the EPICOLON study.
(0.03 MB DOC)

Table S1 MethyLight primers and probes.
(0.04 MB DOC)
