# Supplementary material for: Aberrant Gene Promoter Methylation Associated with Sporadic Multiple Colorectal Cancer
Source: PLoS One. 2010 Jan 19;5(1):e8777. doi: 10.1371/journal.pone.0008777 (PMC2808250; doi:10.1371/journal.pone.0008777)
Supplement: Note S1 — Investigators from the Gastrointestinal Oncology Group of the Spanish Gastroenterological Association who participated in the EPICOLON study. (0.03 MB DOC) [file pone.0008777.s001.doc]

**Note S1.** Investigators from the Gastrointestinal Oncology Group of the Spanish Gastroenterological Association who participated in the EPICOLON study.

Hospital 12 de Octubre, Madrid: Juan Diego Morillas (local coordinator), Raquel Muñoz, Marisa Manzano, Francisco Colina, Jose Díaz, Carolina Ibarrola, Guadalupe López, Alberto Ibáñez; Hospital Clínic, Barcelona: Antoni Castells (local coordinator), Virgínia Piñol, Sergi Castellví-Bel, Francesc Balaguer, Victoria Gonzalo, Teresa Ocaña, María Dolores Giráldez, Maria Pellisé, J. Ignasi Elizalde, Anna Serradesanferm, Josep M. Piqué; Hospital Clínico Universitario, Zaragoza: Ángel Lanas (local coordinator), Javier Alcedo, Javier Ortego; Hospital Cristal-Piñor, Complexo Hospitalario de Ourense: Joaquin Cubiella (local coordinator), Mª Soledad Díez, Mercedes Salgado, Eloy Sánchez, Mariano Vega; Hospital del Mar, Barcelona: Montserrat Andreu (local coordinator), Xavier Bessa, Beatriz Bellosillo, Mar Iglesias, Gemma Navarro, Felipe Bory, Agustín Seoane; Hospital Donosti, San Sebastián: Luis Bujanda (local coordinator), Juan Ignacio Arenas, Isabel Montalvo, Julio Torrado, Ángel Cosme; Hospital General Universitario de Alicante: Artemio Payá (local coordinator), Rodrigo Jover, Juan Carlos Penalva, Cristina Alenda; Hospital General de Granollers: Joaquim Rigau (local coordinator), Ángel Serrano, Anna Giménez; Hospital General de Vic: Joan Saló (local coordinator), Eduard Batiste-Alentorn, Josefina Autonell, Ramon Barniol; Hospital General Universitario de Guadalajara: Ana María García (local coordinator), Fernando Carballo, Antonio Bienvenido, Eduardo Sanz, Fernando González, Jaime Sánchez; Hospital General Universitario de Valencia: Enrique Medina (local coordinator), Jaime Cuquerella, Pilar Canelles, Miguel Martorell, José Ángel García, Francisco Quiles, Elisa Orti; Hospital do Meixoeiro, Vigo: Juan Clofent (local coordinator), Jaime Seoane, Antoni Tardío, Eugenia Sanchez; Hospital San Eloy, Baracaldo: Luis Bujanda (local coordinator), Carmen Muñoz, María del Mar Ramírez, Araceli Sánchez; Hospital Universitari Germans Trias i Pujol, Badalona: Xavier Llor (local coordinator), Rosa M. Xicola, Marta Piñol, Mercè Rosinach, Anna Roca, Elisenda Pons, José M. Hernández, Miquel A. Gassull; Hospital Universitari Mútua de Terrassa: Fernando Fernández-Bañares (local coordinator), Josep M. Viver, Antonio Salas, Jorge Espinós, Montserrat Forné, Maria Esteve; Hospital Universitari Arnau de Vilanova, Lleida: Josep M. Reñé (local coordinator), Carmen Piñol, Juan Buenestado, Joan Viñas; Hospital Universitario de Canarias: Enrique Quintero (local coordinator), David Nicolás, Adolfo Parra, Antonio Martín; Hospital Universitario La Fe, Valencia: Lidia Argüello (local coordinator), Vicente Pons, Virginia Pertejo, Teresa Sala; Hospital Universitario Reina Sofía, Córdoba: Antonio Naranjo (local coordinator), María Dolores Giraldez, María del Valle García, Patricia López, Fernando López, Rosa Ortega, Javier Briceño, Javier Padillo; Fundació Hospital Son Llatzer, Palma de Mallorca: Àngels Vilella (local coordinator), Carlos Dolz, Hernán Andreu.
