# Supplementary material for: Aberrant Gene Promoter Methylation Associated with Sporadic Multiple Colorectal Cancer
Source: PLoS One. 2010 Jan 19;5(1):e8777. doi: 10.1371/journal.pone.0008777 (PMC2808250; doi:10.1371/journal.pone.0008777)
Supplement: Table S1 — MethyLight primers and probes. (0.04 MB DOC) [file pone.0008777.s002.doc]

**Table S1**. MethyLight primers and probes.

| **Gene** | **Forward primer** | **Reverse primer** |
| --- | --- | --- |
| *MGMT1*-Mp | TGTTTTTTTTAGGTTTTCGG | AACTATCCCAACATATCCGA |
| *MGMT2*-Enh | TTTCGGATATGTTGGGATAGTT | AAACGACCCAAACACTCAC |
| *RASSF1A* | TTAGTTTGGATTTTGGGGG | AACCCGTACTTCGCTAACTTT |
| *CDKN2A* | GTTTTTTTAGAGGATTTGAGGGA | CCCTCTAATAACCAACCAACC |
| *SFRP1* | GGTTCGGTCGTAGGAGTT | CTAAATACGCCCCGACTC |
| *TMEFF2* | TCGGGATTTGTATTTTGTT | AACCGCCTCTCGAACTCTA |
| *GATA4* | TTAGGGTCGAGTTGTTGG | CTAAAAACCCCCGAAACTAC |
| *HS3ST2 (3OST2)* | GTAGTTTTCGGAGAAGACGG | AACCCTACGATCGCCTAA |
| *ALUC4* | GGTTAGGTATAGTGGTTTATATTTGTAATTTTAGTA | ATTAACTAAACTAATCTTAAACTCCTAACCTCA |
| **Gene** | **Probe** | **PCR amplicon size** |
| *MGMT1*-Mp | 6FAM-TTACGTCGTTATTTTCGTG | 106 pb |
| *MGMT2*-Enh | 6FAM-TTCGACGTTCGTAGGTT | 108 pb |
| *RASSF1A* | 6FAM-TTCGTTCGGTTCGCGTT | 98 pb |
| *CDKN2A* | 6FAM-TTCGTTAGTATCGGAGGAA | 93 pb |
| *SFRP1* | 6FAM-TGTCGCGCGTTCG | 117 pb |
| *TMEFF2* | 6FAM-TTACGTGATTTCGGGCG | 128 pb |
| *GATA4* | 6FAM-AGGCGGTCGGCGTA | 112 pb |
| *HS3ST2 (3OST2)* | 6FAM-ATTCGCGTGGTCGTG | 87 pb |
| *ALUC4* | 6FAM-CCTACCTTAACCTCCC | 98 pb |
